# Supplementary material for: Concordance between Dash Diet and Hypertension: Results from the Mediators of Atherosclerosis in South Asians Living in America (MASALA) Study
Source: Nutrients. 2023 Aug 17;15(16):3611. doi: 10.3390/nu15163611 (PMC10458588; doi:10.3390/nu15163611)
Supplement: Supplementary file 1 [file nutrients-15-03611-s001.zip › nutrients-2529602-supplementary.pdf]

Supplementary Table S1. Scoring criteria for the DASH-Style diet and mean intake for Q1 (low consumption) and Q5 (high consumption) and median intake among South Asians in the MASALA study (n=716).

Component	Foods	Scoring Criteria	Q1, Servings/d	Q5, Servings/d	Median Intake
Fruit	Apple/Pear; Citrus Fruit; Banana; Grapes; Peach/Nectarine/Plum; Melon; Tropical Fruit	Q1 = 1 point<br>Q2 = 2 points<br>Q3 = 3 points<br>Q4 = 4 points<br>Q5 = 5 points	0.6	4.0	1.8
Vegetables	Bell Peppers; French/String Beans; Broccoli; Cauliflower; Cabbage; Carrots; Celery; Dark Leafy Green Vegetables; Mushrooms; Okra/Bhindi; Onion; Summer Squash/Zucchini; Tomato; Vegetable Kofta; Mixed Vegetable Salad/Stir Fry; Root Vegetables		1.6	7.8	3.6
Whole Grains	Whole Wheat Bread/Roll (100%, incl. dark rye; 60% incl light rye); Roti/Chapati; Bran Granola Cereals; Whole Wheat Cereals; Cooked Cereal (i.e. porridge, oatmeal, dalia, bulgar); Brown Rice; Quinoa		0.6	3.5	1.8
Low-fat Dairy	Milk (including Skim, 1%, 2%, and lactaid); Part-Skim cheese; Low-Fat Plain Yogurt		0.1	2.8	1.1
Nuts and Legumes	Peas/Matar; Lentils; Dal; Sambhar/Rasam; Chickpeas; Other Dried Beans; Nuts; Tofu; Peanut Butter		0.8	3.5	2.0
Sodium[a]	Sum of sodium content of all foods in FFQ	Q1 = 5 points<br>Q2 = 4 points<br>Q3 = 3 points<br>Q4 = 2 points<br>Q5 = 1 point	1,582 mg	4,224 mg	2,553 mg
Sugar-Sweetened Beverages[a]	Non-Diet Cola; Clear Soft Drinks (i.e. 7-up/Sprite); Orange/Grapefruit Juice; Apple/Pineapple Juice; Fruit Drink; Iced Tea; Lemonade; Yogurt Drink (i.e. Lassi with sugar)		0.003	1.4	0.15
Red and Processed Meats[a]	Beef; Pork; Goat/Lamb; Pork Sausage; Ham; Salami; Bologna; Bacon; Liver; Fried Chicken Nuggets		0	0.6	0

[a] Higher quintiles represent higher intake. In constructing the DASH score, higher intake (higher quintiles) of these components receive lower scores

Supplementary Table S2. Age-adjusted and multivariable-adjusted hypertension and blood pressure by DASH group, among South Asian women in the MASALA study (n=322).

	DASH Score 13-20 (lowest) (n=38)	DASH Score 21-28 (n=197)		DASH Score 29-35 (highest) (n=87)		P_{trend}*
	Reference	RRR/IRR/ (SE)	95% CI	RRR/IRR/ (SE)	95% CI	
Incident Hypertension[1]						
Age Adjusted	1.00	1.75 (1.15)	0.49, 6.32	0.58 (0.47)	0.12, 2.81	0.26
Model 1[+]	1.00	1.89 (1.28)	0.50, 7.16	0.66 (0.55)	0.13, 3.34	0.34
Model 2[++]	1.00	1.98 (1.40)	0.49, 7.92	0.70 (0.60)	0.13, 3.71	0.41
Model 3[+++]	1.00	2.06 (0.70)	0.51, 8.37	0.81 (0.70)	0.15, 4.42	0.56
Prevalent Hypertension[1]						
Age Adjusted	1.00	1.61 (0.75)	0.65, 4.02	1.24 (0.63)	0.46, 3.33	0.98
Model 1[+]	1.00	1.93 (0.88)	0.71, 4.71	1.48 (0.77)	0.53, 4.11	0.80
Model 2[++]	1.00	1.41 (0.76)	0.49, 4.03	1.07 (0.61)	0.35, 3.29	0.82
Model 3[+++]	1.00	1.40 (0.75)	0.49, 4.01	1.02 (0.60)	0.32, 3.20	0.75
Systolic Blood Pressure						
Age Adjusted	0.00	-0.65 (3.13)	-6.80, 5.50	-3.72 (3.42)	-10.45, 3.02	0.18
Model 1[+]	0.00	0.03 (3.18)	-6.23, 6.30	-2.64 (3.50)	-9.52, 4.24	0.31
Model 2[++]	0.00	-0.21 (3.21)	-6.52, 6.10	-2.90 (3.50)	-9.79, 3.99	0.28
Model 3[+++]	0.00	-0.34 (3.21)	-6.66, 5.99	-3.34 (3.56)	-10.34, 3.67	0.23

Diastolic Blood Pressure						
Age Adjusted	0.00	-2.19 (1.69)	-5.52, 1.13	-2.50 (1.85)	-6.15, 1.14	0.25
Model 1[+]	0.00	-1.57 (1.72)	-4.95, 1.81	-1.62 (1.89)	-5.33, 2.09	0.50
Model 2[++]	0.00	-1.76 (1.75)	-5.19, 1.67	-1.78 (1.91)	-5.53, 1.97	0.47
Model 3[+++]	0.00	-1.80 (1.75)	-5.24, 1.65	-1.91 (1.94)	-5.73, 1.90	0.42

[1]Incident and Prevalent Hypertension at exam 2, defined by NCEP criterion (130/85 mmHg)
RRR: Relative Risk Ratio for the multinomial logistic regression model; IRR: Incidence Rate Ratio for the negative binomial regression model; SE: Standard Error; CI: Confidence Interval
*p-trend calculated by unadjusted linear regression, using 3 groups of DASH diet score as an ordinal variable
[+]Model 1: Adjusted for age, sex (male/female), percent life lived in the U.S., education (≥Bachelors/<Bachelors), physical activity (ideal, intermediate, poor), smoking (current/former vs. never), alcohol intake (no consumption/any)
[++]Model 2: Model 1 + CAC score category (0, 1-100, 101-400, >400), HDL cholesterol, LDL cholesterol, diabetes, BMI (kg/m^2)
[+++]Model 3: Model 2 + energy (kcal/d)

Supplementary Table S3. Age-adjusted and multivariable-adjusted hypertension and blood pressure by DASH group, among South Asian men in the MASALA study (n=394).

	DASH Score 13-20 (lowest) (n=89)	DASH Score 21-28 (n=247)		DASH Score 29-35 (highest) (n=58)		P_{trend}*
	Reference	RRR/IRR/ (SE)	95% CI	RRR/IRR/ (SE)	95% CI	
Incident Hypertension[1]						
Age Adjusted	1.00	0.88 (0.31)	0.44, 1.76	0.31 (0.18)	0.10, 0.94	0.06
Model 1[+]	1.00	0.66 (0.25)	0.31, 1.40	0.19 (0.12)	0.05, 0.65	**0.01**
Model 2[++]	1.00	0.70 (0.28)	0.31, 1.54	0.23 (0.15)	0.06, 0.83	**0.03**
Model 3[+++]	1.00	0.70 (0.28)	0.31, 1.55	0.23 (0.15)	0.06, 0.82	**0.03**
Prevalent Hypertension[1]						
Age Adjusted	1.00	1.11 (0.33)	0.62, 1.99	0.60 (0.24)	0.27, 1.32	0.28
Model 1[+]	1.00	0.95 (0.31)	0.50, 1.80	0.46 (0.21)	0.19, 1.13	0.12
Model 2[++]	1.00	1.13 (0.41)	0.55, 2.32	0.62 (0.32)	0.23, 1.69	0.42
Model 3[+++]	1.00	1.14 (0.42)	0.56, 2.35	0.67 (0.35)	0.24, 1.87	0.53
Systolic Blood Pressure						
Age Adjusted	0.00	-1.21 (1.93)	-5.00, 2.58	-1.45 (2.63)	-6.62, 3.72	0.54
Model 1[+]	0.00	-1.26 (2.05)	-5.28, 2.76	-1.64 (2.85)	-7.25, 3.97	0.54
Model 2[++]	0.00	-1.09 (2.05)	-5.11, 2.94	-0.99 (2.91)	-6.72, 4.74	0.69
Model 3[+++]	0.00	-1.09 (2.05)	-5.13, 2.94	-1.05 (2.95)	-6.86, 4.75	0.67
Diastolic Blood Pressure						
Age Adjusted	0.00	-0.65 (1.13)	-2.87, 1.58	-1.96 (1.55)	-5.00, 1.08	0.22
Model 1[+]	0.00	-0.68 (1.20)	-3.05, 1.68	-1.95 (1.68)	-5.25, 1.35	0.26

Model 2[++]	0.00	-0.77 (1.21)	-3.14, 1.60	-1.69 (1.72)	-5.07, 1.68	0.32
Model 3[+++]	0.00	-0.77 (1.21)	-3.14, 1.61	-1.69 (1.74)	-5.11, 1.72	0.33

[1]Incident and Prevalent Hypertension at exam 2, defined by NCEP criterion (130/85 mmHg)
RRR: Relative Risk Ratio for the multinomial logistic regression model; IRR: Incidence Rate Ratio for the negative binomial regression model; SE: Standard Error; CI: Confidence Interval
*p-trend calculated by unadjusted linear regression, using 3 groups of DASH diet score as an ordinal variable
[+]Model 1: Adjusted for age, sex (male/female), percent life lived in the U.S., education (≥Bachelors/<Bachelors), physical activity (ideal, intermediate, poor), smoking (current/former vs. never), alcohol intake (no consumption/any)
[++]Model 2: Model 1 + CAC score category (0, 1-100, 101-400, >400), HDL cholesterol, LDL cholesterol, diabetes, BMI (kg/m^2)
[+++]Model 3: Model 2 + energy (kcal/d)

Supplementary Table S4. Age-adjusted and multivariable-adjusted incident and prevalent hypertension (from exam 1 to exam 2), systolic and diastolic blood pressure by DASH component score, among South Asian adults in the MASALA study (n=716).

	Incident Hypertension RRR (95% CI)	Prevalent Hypertension RRR (95% CI)	Systolic Blood Pressure β (95% CI)	Diastolic Blood Pressure β (95% CI)
Fruit				
Lowest Intake (score=1)	1.00 (Reference)		0.00 (Reference)	
Highest Intake (score=5)	**3.43 (1.35, 8.68)**	1.12 (0.56, 2.24)	-1.27 (-5.53, 2.99)	10.83 (-1.59, 3.24)
Vegetable				
Lowest Intake (score=1)	1.00 (Reference)		0.00 (Reference)	
Highest Intake (score=5)	1.01 (0.43, 2.39)	0.87 (0.42, 1.78)	0.16 (-4.29, 4.62)	-0.41 (-2.94, 2.12)
Grains				
Lowest Intake (score=1)	1.00 (Reference)		0.00 (Reference)	
Highest Intake (score=5)	**0.26 (0.11, 0.62)**	0.66 (0.32, 1.34)	1.66 (-2.66, 5.98)	-0.45 (-2.88, 1.99)
Low-Fat Dairy				
Lowest Intake (score=1)	1.00 (Reference)		0.00 (Reference)	
Highest Intake (score=5)	1.54 (0.72, 3.30)	1.28 (0.66, 2.47)	-0.38 (-4.34, 3.57)	-1.67 (-3.91, 0.58)
Nuts/Legumes				
Lowest Intake (score=1)	1.00 (Reference)		0.00 (Reference)	
Highest Intake (score=5)	0.66 (0.28, 1.53)	0.60 (0.29, 1.25)	-2.23 (-6.66, 2.19)	0.97 (-1.54, 3.48)
Reverse Scored DASH Components				
Sodium (mg/d)				
Highest Intake (score=1)	1.00 (Reference)		0.00 (Reference)	
Lowest Intake (score=5)	0.44 (0.13, 1.49)	0.75 (0.27, 2.10)	-3.57 (-9.93, 2.79)	-2.18 (-5.78, 1.42)
Sugar-Sweetened Beverages				
Highest Intake (score=1)	1.00 (Reference)		0.00 (Reference)	
Lowest Intake (score=5)	0.65 (0.29, 1.46)	1.29 (0.64, 2.59)	-1.32 (-5.56, 2.93)	-0.01 (-2.42, 2.39)
Red/Processed Meat				
Highest Intake (score=1)	1.00 (Reference)		0.00 (Reference)	
Lowest Intake (score=5)	0.83 (0.42, 1.64)	0.79 (0.45, 1.41)	0.08 (-3.45, 3.62)	-0.93 (-2.93, 1.07)

Multivariable Model: Adjusted for age, sex (male/female), percent life lived in the U.S., education (≥bachelor's/<bachelor's), physical activity (ideal, intermediate, poor), smoking (current/former vs. never), alcohol intake (no consumption/any), CAC score category (0, 1-100, 101-400, >400), HDL cholesterol, LDL cholesterol, diabetes, BMI (kg/m^2), energy intake (kilocalories/day)
